# Prenatal Diagnosis of Congenital High Airway Obstruction Syndrome: Report of Two Cases and Brief Review of the Literature

**DOI:** 10.1155/2013/728974

**Published:** 2013-10-22

**Authors:** Burcu Artunc Ulkumen, Halil Gursoy Pala, Nalan Nese, Serdar Tarhan, Yesim Baytur

**Affiliations:** ^1^Celal Bayar University, School of Medicine, Obstetrics and Gynecology Department, Manisa, Turkey; ^2^Celal Bayar University, School of Medicine, Pathology Department, Manisa, Turkey; ^3^Celal Bayar University, School of Medicine, Radiology Department, Manisa, Turkey

## Abstract

Congenital high airway obstruction syndrome (CHAOS) is the obstruction of the fetal upper airways, which may be partial or complete. It is usually incompatible with life. Prenatal recognition of the disease is quite important due to the recently described management options. We report here two cases of CHAOS due to tracheal atresia diagnosed by antenatal ultrasonography and fetal MRI. We also briefly review the relevant literature with the associated management options.

## 1. Introduction

Congenital high airway obstruction syndrome (CHAOS) is defined as complete or partial obstruction of the fetal upper airways. This clinical condition was brought into notice firstly by Hedrick in the late 1900s [1]. CHAOS is usually caused by atresia or stenosis of the larynx or trachea. The true incidence of CHAOS is unknown. If the syndrome is unrecognized during the prenatal period, it usually results in stillbirth or death shortly after delivery [2]. Fortunately, more cases can be recognized in utero nowadays, as there are significant technical improvements in prenatal imaging. Bilaterally enlarged hyperechoic lungs, dilated airways, and flattened or inverted diaphragm are the typical prenatal sonographic findings. Fetal ascites and nonimmune hydrops may also be associated with the clinical condition [3]. Due to the recently described management options, prenatal definition of fetal airway obstruction has come into prominence with the hope of neonatal outcome improvements [4]. We report here two cases of CHAOS due to tracheal atresia diagnosed by antenatal ultrasonography and fetal MRI. 

## 2. Case 1

**Figure 1:**
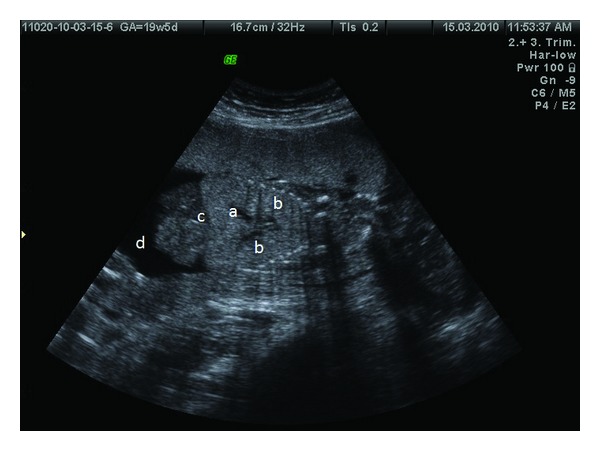
Antenatal sonography of case 1 showing dilated main bronchi (a), large hyperechoic lungs (b), inverted diaphragm (c), and ascites (d).

**Figure 2:**
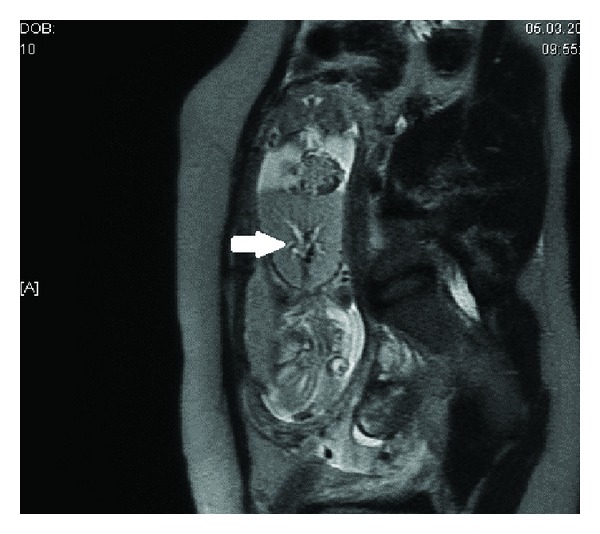
Antenatal MRI of case 1 showing dilated airways (arrow), hyperxpanded lungs, inverted diaphragm, and ascites.

A 31-year-old woman with a previous live birth was referred for a routine second trimester antenatal ultrasound at 19-week gestational age. Ultrasound examination revealed that the fetus had bilateral large echogenic lungs. The principal bronchi appeared dilated (Figure 1). The diaphragm was inverted. The heart was centrally placed and seemed to be compressed by the enlarged lungs, and there was moderate ascites. However, amniotic fluid was normal. An MRI study was performed which again showed bilateral large and distinctly hyperintense lungs. Significantly, dilated main bronchi could be clearly seen. Inversion of the diaphragm, the typical small compressed fetal heart, and ascites were also confirmed and no additional anomaly could be found (Figure 2).

**Figure 3:**
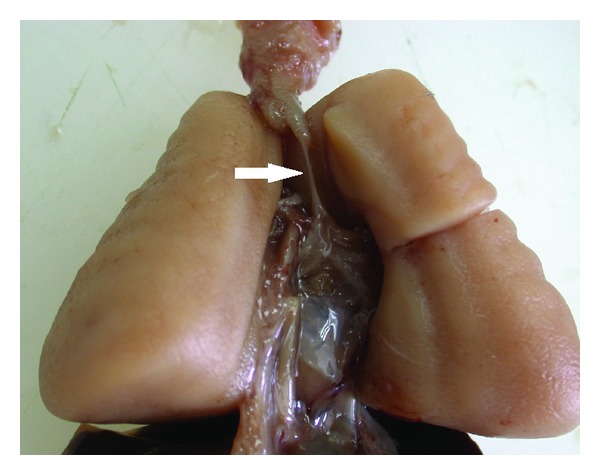
Postmortem autopsy findings of case 1 compatible with tracheal atresia (arrow): voluminous lungs and dilated upper airways.

Based on ultrasound and MRI findings, diagnosis of CHAOS due to tracheal atresia was made. The parents were counselled regarding the relatively poor prognosis of the syndrome, and the pregnancy was terminated according to the choice of the family. The fetus had severe hydrops manifested by ascites and pericardial and pleural effusions. Postmortem examination was compatible with a complete tracheal atresia as the cause of airway obstruction (Figure 3). The lungs of this fetus were very enlarged (Figure 3). 

## 3. Case 2

**Figure 4:**
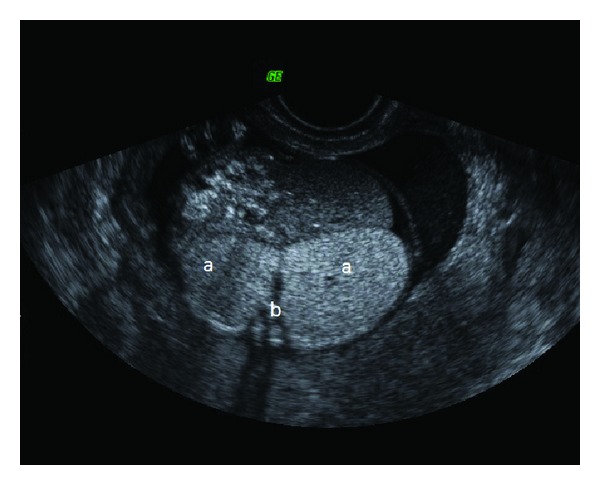
Antenatal sonography of case 2 showing large hyperechoic lungs (a), dilated main bronchi (b), large hyperechoic lungs, inverted diaphragm, and ascites.

A 24-year-old primiparous woman presented for a routine second trimester antenatal ultrasound at 18-week gestational age. Ultrasound examination showed that the fetus had bilateral large hyperechoic lungs. The diaphragm was inverted. The heart seemed small and was centrally displaced. There was massive ascites. The tracheobronchial tree appeared dilated (Figure 4). However, amniotic fluid quantity was normal. 

**Figure 5:**
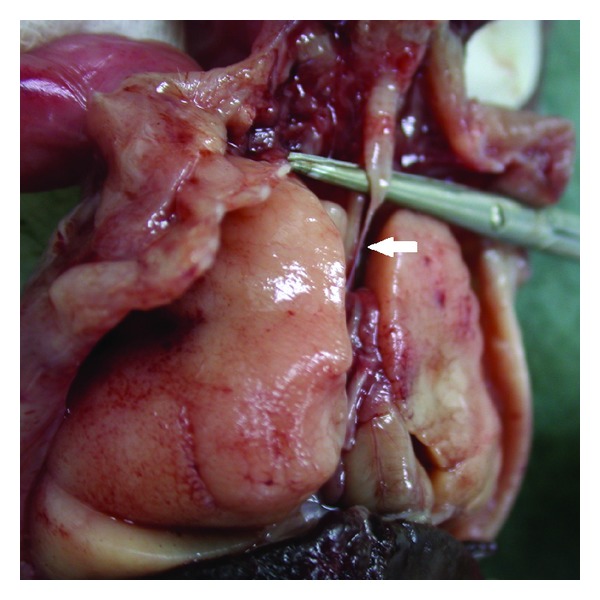
Postmortem autopsy findings of case 2 compatible with tracheal atresia (arrow): voluminous lungs and dilated upper airways.

Based on ultrasound findings, diagnosis of CHAOS was made. The parents were counselled regarding the relatively poor prognosis, and the EXIT procedure was offered. However, the parents decided to get an elective termination, and the pregnancy was terminated. The fetus had severe hydrops manifested by anasarca and pericardial and pleural effusions. Postmortem examination was compatible with a complete tracheal atresia as the cause of airway obstruction (Figure 5). The lungs of this fetus were significantly enlarged (Figure 5). 

## 4. Discussion

Tracheal atresia is a very rare congenital malformation which takes place by deficient recanalization of the upper airways around the 10th week of gestation resulting in a clinical spectrum defined as congenital high airway obstruction syndrome (CHAOS) [1]. In a healthy fetus, the fluid secreted by fetal lung is absorbed through the tracheobronchial tree. However, in case of any obstruction in the tracheobronchial tree, this fluid cannot be cleared. The accumulation of the fetal lung fluid results in gradual increase of intratracheal pressure leading to enlargement of the lungs. It is the beginning of a chain reaction: the enlarged lungs cause compression of the heart and great veins. Due to the compression, the heart replaces centrally and becomes small and dysfunctional. Decreased venous return and dysfunctional cardiovascular system end in ascites and hydrops. The diaphragm flattens or inverts according to the severity of the process [2]. Besides tracheal atresia, the other rare underlying causes of CHAOS are laryngeal agenesis, subglottic stenosis or atresia, and laryngeal webs or cysts. However, the obstruction is mostly secondary to laryngeal atresia [1, 4]. 

The main diagnostic tool for prenatal diagnosis of CHAOS is sonography which has typical findings on evaluation. As a natural conclusion of the pathological process, bilateral large hyperechoic lungs, small, compressed, and centrally replaced heart, flattened or inverted diaphragm, and ascites are characteristic findings on sonographic examination [3]. Regarding the amniotic fluid index, compression of the esophagus by dilated airways may lead to polyhydramnios, as the fetal swallowing of the fluid is disrupted. On the other hand, impaired swallowing of the fetus may also cause oligohydramnios [2]. We think that the amniotic fluid quantity is not a constant marker for the diagnosis, as it is remarkable that both of our cases had normal amniotic fluid index. The gestational age at the diagnosis may affect the amniotic fluid quantity. Polyhydramnios may not be present due to the examination in the early 2nd trimester in our cases. 

The typical sonographic findings can also be recognized on MRI including voluminous lungs, centrally displaced small heart, inverted diaphragm, and ascites. Sonography is first-line diagnostic imaging tool due to its low cost and widespread use. However, especially if any fetal surgical intervention is planned, MR imaging can be used additionally by following the dilated airway up to the level of obstruction, as it is more effective for detecting the exact level of obstruction [5]. 

CHAOS is most often misdiagnosed as bilateral congenital cystic adenomatoid malformation (CCAM) [1]. CCAM (especially type III) and upper airway obstruction secondary to intrinsic causes such as tracheal or laryngeal atresia or stenosis and tracheal webs similarly have bilateral uniform hyperechogenic appearance of the fetal lungs on sonographic examination [6]. In order to make a differentiation between CHAOS and CCAM type III, the obstruction site with distal airway dilatation (present in CHAOS) and the systemic arterial supply (present in CCAM type III) must be clearly seen.

Congenital high airway obstruction syndrome should be also differentiated from extrinsic causes of tracheolaryngeal obstruction. Some of these extrinsic causes are lymphatic malformation, cervical teratoma, and vascular rings like double aortic arch [1, 3].

CHAOS is mostly sporadic, and the exact incidence is not known [1, 2, 7]. In utero death of the affected cases; being a part of some genetic syndromes; better detection rate of the anomalies by the means of technical improvement of imaging tools; and data only from isolated cases instead of studies consisting of large series may explain this indefinable incidence. The most common associated genetic disorder with CHAOS is Fraser's syndrome which is inherited by autosomal recessive form and characterized by urogenital defects, laryngeal atresia, syndactyly, and cryptophthalmos [2]. CHAOS may be also a part of Cri-du-Chat syndrome, short-rib polydactyly syndrome, and velocardiofacial syndrome [2, 8]. Apart from this, a case of CHAOS with autosomal dominant inheritance of the father and his two affected children was reported [7]. The point to take into consideration is the necessity of a detailed evaluation of all CHAOS suspected cases due to possibility of coexistence of any genetic syndrome and significant implications of inheritance for future pregnancies [8].

In the past, CHAOS was thought to be equivalent to a certain fetal death. However, nowadays, especially if CHAOS due to incomplete obstruction is diagnosed in the late 2nd or in the 3rd trimester and if severe hydrops has not occurred yet, the EXIT procedure (ex utero intrapartum treatment) can be offered. The common objective of the procedure is to settle an intact airway for the baby before the fetomaternal circulation is stopped [9]. 

There are few cases with spontaneous antenatal improvements. This progress may be due to spontaneous perforation or tracheoesophageal fistula with the refining of the obstructed fluid leading to decrease in the pressure of airways and reversal of the process [10]. 

As a result, CHAOS is a rare and fatal cause of congenital airway obstruction if unrecognized during prenatal period. Antenatal sonographic imaging shows typical findings which can lead to a diagnosis. MRI is superior to sonography in demonstrating the level of obstruction and in assisting in the differential diagnosis by excluding extrinsic causes of obstruction. This is important especially if any fetal intervention is considered.

## References

[1] Roybal JL, Liechty KW, Hedrick HL (2010). Predicting the severity of congenital high airway obstruction syndrome. *Journal of Pediatric Surgery*.

[2] Joshi P, Satija L, George RA (2012). Congenital high airway obstruction syndrome-antenatal diagnosis of a rare case of airway obstruction using multimodality imaging. *Medical Journal Armed Forces India*.

[3] Garg M (2008). Case report: antenatal diagnosis of congenital high airway obstruction syndrome. Laryngeal atresia. *Indian Journal of Radiology and Imaging*.

[4] Courtier J, Poder L, Wang ZJ, Westphalen AC, Yeh BM, Coakley FV (2010). Fetal tracheolaryngeal airway obstruction: prenatal evaluation by sonography and MRI. *Pediatric Radiology*.

[5] Coakley FV, Hricak H, Filly RA, Barkovich AJ, Harrison MR (1999). Complex fetal disorders: effect of MR imaging on management: preliminary clinical experience. *Radiology*.

[6] King SJ, Pilling DW, Walkinshaw S (1995). Fetal echogenic lung lesions: prenatal ultrasound diagnosis and outcome. *Pediatric Radiology*.

[7] Vanhaesebrouck P, De Coen K, Defoort P (2006). Evidence for autosomal dominant inheritance in prenatally diagnosed CHAOS. *European Journal of Pediatrics*.

[8] Lim F-Y, Crombleholme TM, Hedrick HL (2003). Congenital high airway obstruction syndrome: natural history and management. *Journal of Pediatric Surgery*.

[9] Walker P, Cassey J, O’Callaghan S (2005). Management of antenatally detected fetal airway obstruction. *International Journal of Pediatric Otorhinolaryngology*.

[10] Kuwashima S, Kitajima K, Kaji Y, Watanabe H, Watabe Y, Suzumura H (2008). MR imaging appearance of laryngeal atresia (congenital high airway obstruction syndrome): unique course in a fetus. *Pediatric Radiology*.

